# Preliminary Acceptability of a Home-Based Peripheral Blood Collection Device for Viral Load Testing in the Context of Analytical Treatment Interruptions in HIV Cure Trials: Results from a Nationwide Survey in the United States

**DOI:** 10.3390/jpm12020231

**Published:** 2022-02-07

**Authors:** Karine Dubé, Shadi Eskaf, Elizabeth Hastie, Harsh Agarwal, Laney Henley, Christopher Roebuck, William B. Carter, Lynda Dee, Jeff Taylor, Derrick Mapp, Danielle M. Campbell, Thomas J. Villa, Beth Peterson, Kenneth M. Lynn, Linden Lalley-Chareczko, Emily Hiserodt, Sukyung Kim, Daniel Rosenbloom, Brad R. Evans, Melanie Anderson, Daria J. Hazuda, Lisa Shipley, Kevin Bateman, Bonnie J. Howell, Karam Mounzer, Pablo Tebas, Luis J. Montaner

**Affiliations:** 1 UNC Gillings School of Global Public Health, University of North Carolina at Chapel Hill, Chapel Hill, NC 27599, USA; hagarwal@unc.edu (H.A.); laneyh1999@gmail.com (L.H.); 2 Independent Public Health Researcher and Consultant, Chapel Hill, NC 27516, USA; shadieskaf@gmail.com; 3 School of Medicine, University of California San Diego, La Jolla, CA 92093, USA; ehastie@health.ucsd.edu; 4 Department of Science and Technology Studies, Cornell University, Ithaca, NY 14850, USA; croebuck@berkeley.edu; 5 Martin Delaney BEAT-HIV Collaboratory Community Advisory Board (CAB), Philadelphia, PA 19104, USA; benjam1229@gmail.com; 6 AIDS Action Baltimore, Baltimore, MD 21202, USA; lyndamdee@aol.com; 7 Delaney AIDS Research Enterprise (DARE) Community Advisory Board (CAB), San Francisco, CA 94110, USA; jeff.taylor@harp-ps.org (J.T.); ms.danielle.campbell@gmail.com (D.M.C.); 8 AIDS Treatment Activists Coalition (ATAC), Denver, CO 80209, USA; dmapp@shanti.org; 9 HIV + Aging Research Project-Palm Springs (HARP-PS), Palm Springs, CA 92264, USA; 10 Shanti Project, San Francisco, CA 94109, USA; 11 HOPE Martin Delaney Collaboratory, San Francisco, CA 94612, USA; tomvilla2@comcast.net; 12 BELIEVE Martin Delaney Collaboratory, Washington, DC 10021, USA; 13 National HIV & Aging Advocacy Network, Washington, DC 20005, USA; 14 Wistar Institute, Martin Delaney BEAT-HIV Collaboratory, Philadelphia, PA 19104, USA; bpeterson@wistar.org (B.P.); montaner@wistar.org (L.J.M.); 15 Hospital of the University of Pennsylvania, Philadelphia, PA 19107, USA; kelynn@pennmedicine.upenn.edu (K.M.L.); sukyung.kim@pennmedicine.upenn.edu (S.K.); karam.mounzer@pennmedicine.upenn.edu (K.M.); pablo.tebas@pennmedicine.upenn.edu (P.T.); 16 Philadelphia FIGHT Community Health Centers, Philadelphia, PA 19107, USA; lchareczko@fight.org (L.L.-C.); ehiserodt@fight.org (E.H.); 17 Merck & Co, Inc., Kenilworth, NJ 07033, USA; daniel.rosenbloom@merck.com (D.R.); brad.evans@merck.com (B.R.E.); melanie_anderson@merck.com (M.A.); daria_hazuda@merck.com (D.J.H.); lisa_shipley2@merck.com (L.S.); kevin_bateman@merck.com (K.B.); bonnie_howell@merck.com (B.J.H.)

**Keywords:** home-based viral load, acceptability, HIV cure research, analytical treatment interruptions, people with HIV, personalized medicine

## Abstract

Frequent viral load testing is necessary during analytical treatment interruptions (ATIs) in HIV cure-directed clinical trials, though such may be burdensome and inconvenient to trial participants. We implemented a national, cross-sectional survey in the United States to examine the acceptability of a novel home-based peripheral blood collection device for HIV viral load testing. Between June and August 2021, we distributed an online survey to people with HIV (PWH) and community members, biomedical HIV cure researchers and HIV care providers. We performed descriptive analyses to summarize the results. We received 73 survey responses, with 51 from community members, 12 from biomedical HIV cure researchers and 10 from HIV care providers. Of those, 51 (70%) were cisgender men and 50 (68%) reported living with HIV. Most (>80% overall) indicated that the device would be helpful during ATI trials and they would feel comfortable using it themselves or recommending it to their patients/participants. Of the 50 PWH, 42 (84%) indicated they would use the device if they were participating in an ATI trial and 27 (54%) also expressed a willingness to use the device outside of HIV cure studies. Increasing sensitivity of viral load tests and pluri-potency of the device (CD4 count, chemistries) would augment acceptability. Survey findings provide evidence that viral load home testing would be an important adjunct to ongoing HIV cure-directed trials involving ATIs. Survey findings may help inform successful implementation and uptake of the device in the context of personalized HIV care.

## 1. Introduction

Analytical treatment interruptions (ATIs) have become a common feature of HIV cure-directed clinical trials [1,2]. In these investigations, people with HIV (PWH) interrupt antiretroviral treatment (ART) to test whether experimental interventions can keep HIV suppressed in the absence of therapy. Despite ongoing research [3,4,5], there is currently no biomarker available that can accurately predict when virus will return for a given participant. Frequent viral load testing during ATIs is necessary to detect viral rebound and determine when participants must restart ART [1]. Up to two viral load tests per week are necessary, oftentimes translating into high burden and inconvenience for HIV cure trial participants who have to visit the research site multiple times each week [6].

Merck & Co., Inc., Kenilworth, NJ, USA, and Tasso, Inc., Seattle, WA, USA [7] have been developing a home-based viral load collection device that would make ATIs less burdensome and more acceptable for PWH [1,6]. For the current device prototype, blood samples are collected at home, although testing must occur in the laboratory. Using capillary action (no venipuncture), the device allows a minimum of 80 µL of whole, liquid blood to be self-collected in about five minutes. In an international survey of PWH (*n* = 442), 59% of respondents expressed willingness to undergo ATIs if home-based viral load testing were available [8]. Importantly, the ability to self-test for viral load on demand would translate into more personalized monitoring and care and permit ATI trial participants to determine when they become viremic, reducing the risk of unintended HIV transmission to sexual partners [9,10,11,12].

The ongoing COVID-19 pandemic has further precipitated increased appreciation towards home-testing technologies to reduce the risk of SARS-CoV-2 exposure in clinic settings and facilitate self-testing [13]. Previous studies related to HIV [14,15,16,17,18,19] and sexually transmitted infections (STIs) [20,21,22,23,24,25,26,27,28,29] have demonstrated successful use of self-specimen collection mechanisms. Other successful examples of self-specimen collection outside of HIV and STI management include tuberculosis [30], hypertension and cardiovascular health [31,32] and diabetes (in existence since the 1970s) [33]. Acceptability of home-based pre-exposure prophylaxis (PrEP) monitoring also has been shown recently in the United States [34].

To assess the acceptability of the current version of home-based blood collection devices for viral load testing among a diverse group of individuals involved in HIV cure research, we implemented a nationwide, cross-sectional survey in the United States. The primary objective of this survey was to generate preliminary descriptive data and inform the successful implementation and uptake of the home-based viral load testing in the context of ATI trials and eventually personalized HIV care.

## 2. Materials and Methods

### 2.1. Study Design

We conducted a nationwide, cross-sectional, online survey among individuals involved in HIV cure research (e.g., PWH and community advocates, biomedical HIV cure researchers, HIV care providers) in the United States to explore acceptability of home-based peripheral blood collection devices for viral load testing in the context of ATIs. Since our study was framed as a preliminary descriptive study and due to time and resource constraints, our initial minimum sample size was established at 50 respondents, which would yield a margin of error of 14% at a 95% confidence level. All survey respondents provided informed consent and the study was approved by the University of North Carolina at Chapel Hill Institutional Review Board (IRB) in Chapel Hill, NC, USA (# 20-2957).

### 2.2. Recruitment

Between June and August 2021, we recruited participants using known networks focused on HIV cure-directed research in the United States. These included the Martin Delaney Collaboratories Towards an HIV-1 Cure, AIDS Treatment Activists Coalition (ATAC), IBT-Cure Listserv, Centers for AIDS Research (CFAR) and healthcare centers serving PWH—e.g., Philadelphia FIGHT Community Health Centers (fight.org). We did not advertise the survey on social media (e.g., Facebook, Instagram, or Twitter) to expressly avoid bots [35] and increase assurance in the validity of the answers received. To be eligible for the survey, respondents had to be currently involved in HIV cure research, ≥18 years old and living in one of the 50 U.S. states or territories. Participants needed to be able to complete the survey in English and share their opinion. There was no exclusion criterium. Self-reported PWH and community members were given the option to participate in a draw to receive a Virtual Incentives [36] gift card of USD 20 per 10 PWH/community members.

### 2.3. Data Collection

We developed the survey instrument in close collaboration with members of the BEAT-HIV Community Advisory Board and Social Sciences Working Group. In addition, ATAC community members (L.D., J.T., D.M. and D.M.C.) reviewed the survey instrument. We programmed the survey database using Qualtrics software (Provo, UT) and participants were free to skip any questions. We extensively pilot-tested the online survey with seven BEAT-HIV- and ATAC-affiliated community members prior to launch.

**Figure 1:**
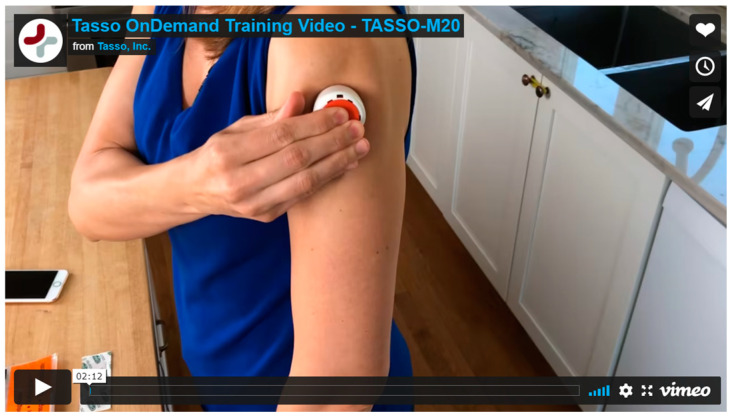
Tasso home-based viral load test device demonstration video (available at https://www.tassoinc.com/tasso-m20-video—accessed on 29 November 2021).

This was an auto-administered, semi-structured survey that included a mix of multiple-choice questions and open-ended questions inviting custom text answers. Following eligibility checking and a robust bot control step, the survey provided a brief orientation about the study and the device. ATIs were described as follows: “Participants are asked to temporarily pause their ART. This is called ATI. The amount of virus in the blood (viral load) will be monitored regularly while they are off their meds by a new method of blood collection”. We defined the device as follows: “The instrument for collecting the blood explained in the demonstration video”. Respondents were instructed to watch a brief demonstration video on the Tasso blood collection device (Figure 1). Respondents were also directed to an IRB-approved version of the device use instructions.

We collected demographic information from all respondents (e.g., gender, sex at birth, age, ethnicity, education and state of residency). We then asked respondents about the nature of their involvement in HIV cure research and if they were a PWH or community member, biomedical HIV cure researcher, HIV care provider, or other involvement.

All respondents subsequently answered a series of survey questions requesting their general feedback about the home-based blood collection device for viral load testing. These included suggestions to improve the device, questions they had about the device, excitement to use the device and concerns about the device. Respondents also provided feedback on the use of the device as part of ATI trials, including the comfort level with using or recommending the device, whether the device should be used as part of ATI trials, frequency of use, desired sensitivity level of the viral load test (e.g., how good the test should be) and device use at home versus medical setting. The subsequent survey section focused on feedback related to mailing blood samples to a laboratory or research site, including comfort level with sending blood through the mail, preferred shipment methods, travel times and ease of mailing samples. Moreover, respondents were offered an opportunity to provide feedback on communicating viral load test results to ATI trial participants and technical support services. Self-reported PHW were asked to answer additional survey questions inquiring about the use of the device in the context of ATI trials and outside of ATI trials and whether the device would affect anxiety, stigma and other social risks for PWH. The survey instrument can be made available upon request by contacting the lead author (K.D.).

### 2.4. Data Analyses

We conducted descriptive analyses to summarize the results. We categorized the participants into three mutually exclusive groups: (1) PWH and community members/advocates (“community”), (2) biomedical HIV cure researchers (“biomedical”) and (3) HIV care providers (“provider”). We used Excel software (version 2018) to conduct all analyses for the closed-ended data and create tables and graphs. Given the small number of respondents, exploratory nature of the survey and skewed samples (towards the “community” group), we did not conduct bivariate nor multivariate analyses. We analyzed the open-ended data using conventional content analyses [37].

## 3. Results

### 3.1. Survey Respondents

There were 73 survey respondents, including 51 PWH/community members, 12 biomedical HIV cure researchers and 10 HIV care providers from across the United States (Table 1). The majority (70%) were cisgender men, White/Caucasian (67%) and non-Hispanic/Latinx (90%). Close to one fifth (22%) of PWH/community respondents were Black/African American. There were 50 (68%) respondents who reported living with HIV, most of whom were also community members. Of these, ten (or one-fifth of PWH) had previously participated in an HIV cure study with an ATI.

### 3.2. General Feedback on the Home-Based Viral Load Test Device from All Participants

After viewing the embedded demonstration video, one-fifth (21%) of respondents had questions about the device. Questions centered around the device’s mechanism of action (capillary), level of pain or discomfort involved, use of the device on other body parts (e.g., legs instead of upper arms), possible device malfunctions, temperature requirements and turnaround time for receiving viral load test results.

Most (95%) respondents provided their initial thoughts about the device. Key categories included excitement (e.g., “I love it!”, “Wonderful idea”), user-friendliness (e.g., “Device looks easy to use and straightforward”, “It looks to be simple and painless to use”) and convenience (e.g., “Especially for people who have a hard time with transportation or have restrictions on mobility”). Respondents compared the device with glucometers used to monitor diabetes and other medical devices (e.g., “Like an auto-injector for migraine medicine I’ve used before”). A biomedical HIV cure researcher noted the urgency of making the device widely available.

*This Is Urgently Needed. Especially with COVID*.

(biomedical HIV Cure Researcher)

Approximately one-fifth (19%) of respondents provided suggestions to improve the device. These included labeling or numbering device parts, adding suction rather than relying solely on capillary flow, immediate testing without mailing blood, lighting system to indicate when the device has filled up, reducing the number of steps and decreasing the environmental footprint. Several respondents desired using the device for other blood tests besides viral load (e.g., CD4 count—discussed further below). A community member commented the following:

*The appeal of the device comes from elimination of a visit to the clinic/lab, so the ability to collect sufficient specimen for all required testing is essential to [the] success of the device*.

(community member)

The majority (70%) reported being excited about the device. Reasons included the innovative nature of the device (e.g., “It’s a step towards more options for home-testing”), device increasing accessibility of viral load testing, sense of control (e.g., “I like the autonomy of self-testing”, “The device enables a patient to take responsibility and ownership of their health status while participating in a trial”), not having to go to the clinic and time-saving aspects. The lack of venipuncture was another perceived advantage. For example, an HIV care provider commented,

*I’m excited about it for my patients that refuse to do labs due to severe needle phobia. This seems like a device we could use in clinic, attach it on at the beginning of the visit while we’re talking/doing exams, and collect the blood at the end*.

(HIV care provider)

However, 11% of respondents were not excited about the device. Reasons included worry about hurting oneself, device not replacing clinic lab work and preference for immediate viral load test results (such as the HIV rapid antibody self-test).

Nevertheless, around half (47%) of respondents identified possible concerns. These were possible side effects such as discomfort or bruising, blood aversion, lower accuracy of viral load test results, hygiene requirements, biohazard disposal at home and risks of infection or contamination. Additional concerns raised included the device not filling up, dexterity requirements, user errors or users not properly following instructions. Respondents also noted possible transportation concerns, mailing issues (discussed further below), turnaround time for viral load test results and possible social risks, such as the theoretical concerns of people getting tested against their will.

Approximately one-fifth (19%) of respondents identified possible privacy or other issues around storing the device at home. These encompassed loss of confidentiality, accidental or inadvertent HIV disclosure, blood spills and device looking similar to a toy resulting in accidental needlesticks or injuries for children.

### 3.3. Feedback on Use of the Device from All Participants

**Figure 2:**
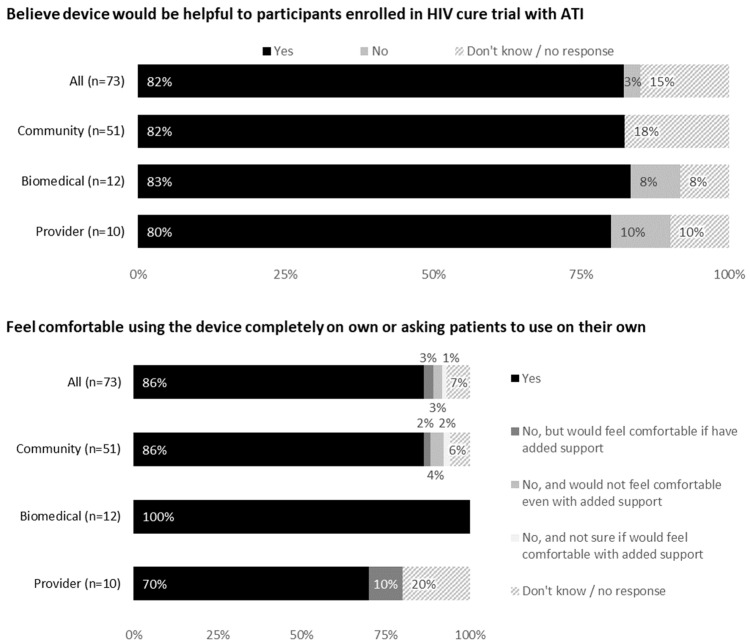

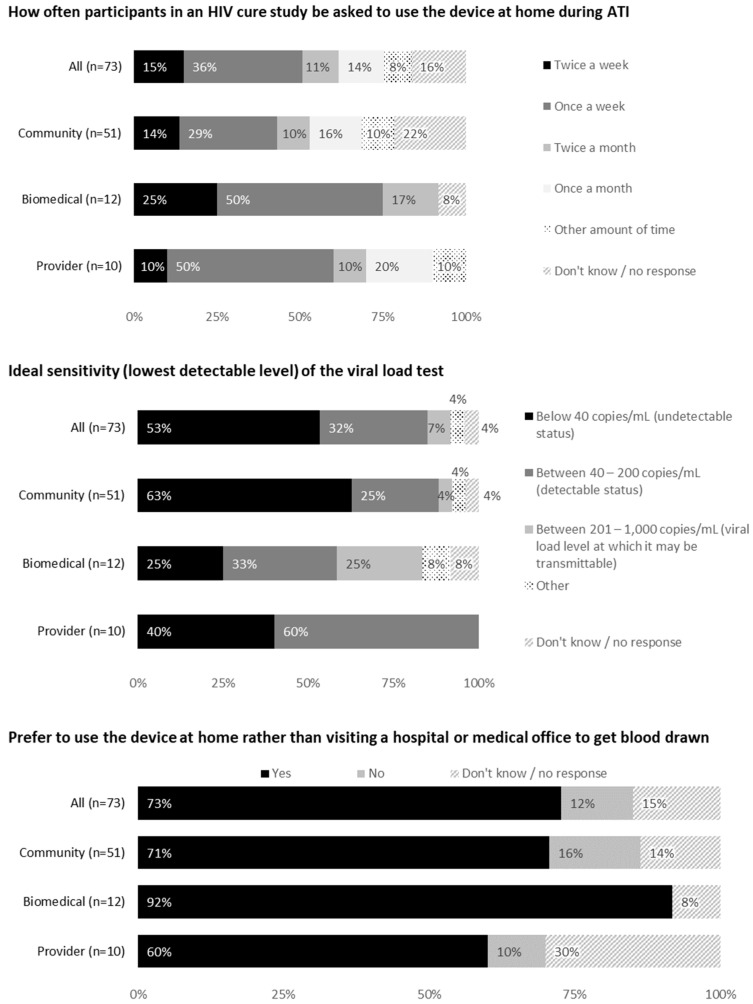
Feedback on use of the device from all participants (United States, 2021).

Most respondents (86% community members, 100% biomedical HIV cure researchers and 70% HIV care providers) indicated they would feel comfortable using the device or asking patients/participants to use it on their own (Figure 2). Reasons for not feeling comfortable included blood or needle aversion and need for additional orientation and support.

Most respondents (82% community members, 83% biomedical HIV cure researchers and 80% HIV care providers) indicated the device would be helpful during an ATI trial. Reasons included scientific reasons (e.g., quicker viral rebound detection), safety reasons (increased self-viral load monitoring, ART restart determination, risk mitigation for onward HIV prevention during ATI, prevention of nosocomial infections such as COVID-19) and reduced participant burden with fewer clinic visits. Psychosocial reasons included peace of mind, sense of self-efficacy and shared participant involvement with the ATI trial.

Exemplary quotes around the urgency of a home-based viral load test device in the ATI context are as follows:

*It would be beneficial because they [participants] can do it [viral load] at home. I know if I was having an ATI, the last place I would want to be is in a hospital where I am potentially exposed to other viruses. I would want to protect all the T-cells I have if I am off my meds and this seems to be an efficient way of stress reduction from participating in a study where I am not being protected from my HIV meds*.

(community member)

*This is urgently needed for our HIV cure projects involving treatment interruption with the associated monitoring. It would be a game changer for both participant safety and convenience*.

(biomedical HIV cure researcher)

*This device would ultimately promote our ability to conduct ATIs as safely as possible. First, it would remove the need for safety-only blood draws to take place in the research center. This means that participants could test from the safety of their home without having to incur the COVID risk of traveling to a research site. Second, it would be very convenient and allow participants to travel while participating in a prolonged*.

*ATI without having to make local arrangements for safety testing. This would increase the acceptability of the monitoring for many participants*.

(biomedical HIV cure researcher)

*This could help avoiding problems with [the] ATI regarding potential transmission to partners, but it should be done in combination with other strategies such as free PrEP to partners or other methods to prevent potential transmission during ATI*.

(biomedical HIV cure researcher)

However, 8% of biomedical researchers and 10% of HIV care providers did not believe the device would be helpful during ATIs. The reasons given were increased availability of local labs for blood draws, need for reliable testing and additional blood work requirements besides viral load.

Over one-third (36%) of respondents would prefer using the device once weekly during an ATI, 15% would prefer twice weekly, 14% would prefer once monthly and 11% would prefer twice monthly. Additional frequencies provided in open-text fields included “as often as needed for the study”, “2 weeks after ATI then monthly” and “pending on the criteria set for the study and participants”.

Most respondents (53%) indicated that the ideal sensitivity of the viral load test should be below 40 copies/mL. Close to one-third (32%) were comfortable with a sensitivity between 40 and 200 copies/mL. Two community members commented as follows:

*Depends on the purpose of the study. For participant safety, I would recommend at least at 40 copies. If [the] study is looking at efficacy, then below, because any VL [viral load] would be important to note in that instance*.

(community member)

*Depends on the study criteria. Probably lower is better to know exactly when virus rebounds. If only to prevent transmission then 200–1000 [copies/mL] would be fine*.

(community member)

Perceived facilitators to using the device provided in the open-text fields included clear orientation in addition to the demonstration video, detailed instructions, oversight at initial use, practice, support if needed, peer educators, mailing device to participants, pre-paid return shipping, reliable test results, rapid turnaround for test results, calendar or text reminders, telemedicine, reasonable price and insurance coverage, and environmental friendliness (e.g., recyclable materials).

Perceived barriers to using the device included pain or discomfort, limited dexterity or cognitive impairment, micro-needle aversion, fear of self-medical procedures, privacy issues in shared living arrangements, limited transportation or mobility, sanitary limitations and device malfunction.

### 3.4. Feedback on Mailing Blood Samples to Laboratory or Research Site

**Figure 3:**
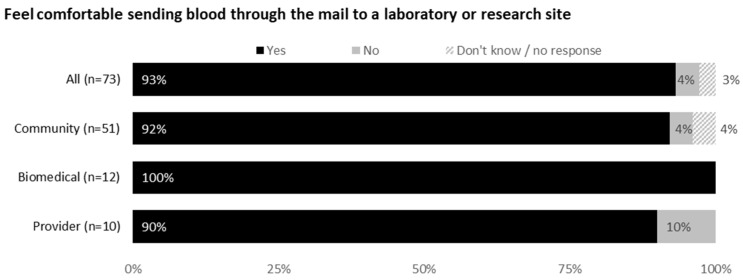

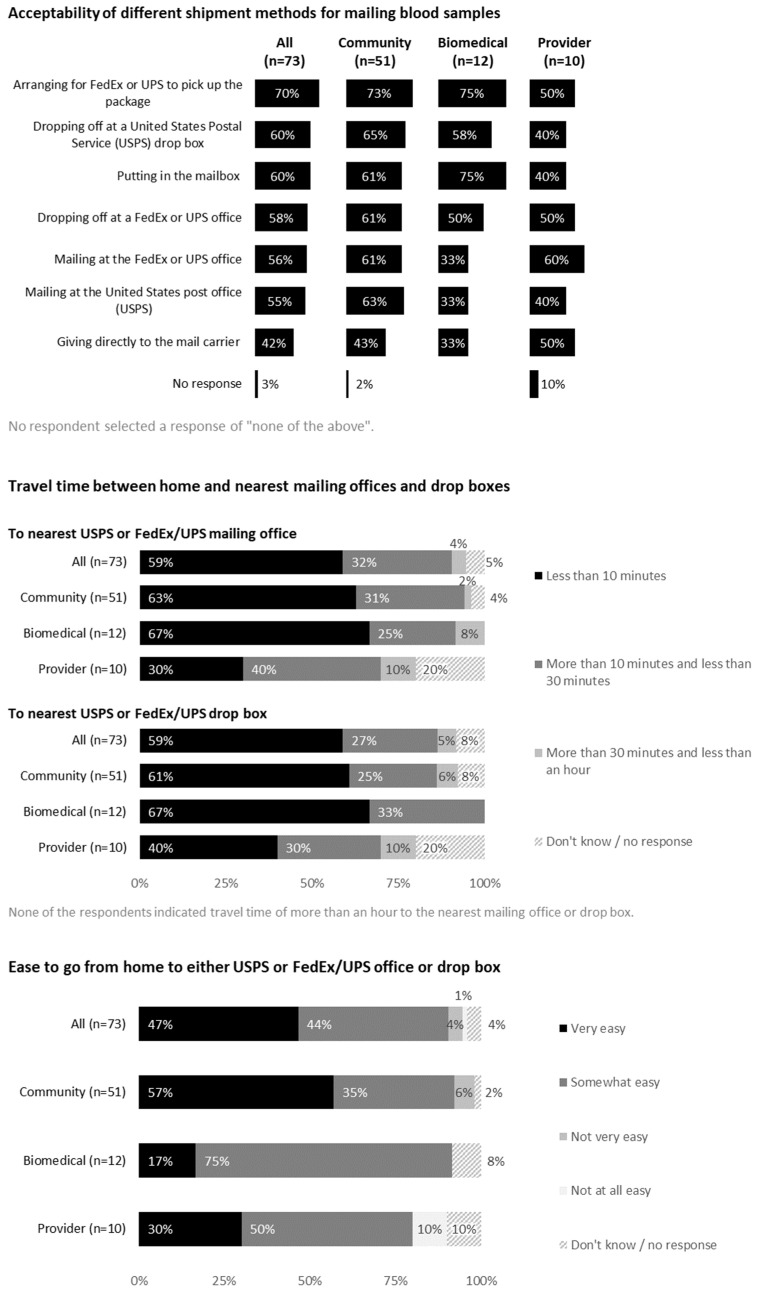
Feedback on mailing blood samples to a laboratory or research site from all participants (United States, 2021).

Most respondents (92% community members, 100% biomedical HIV cure researchers and 90% HIV care providers) would feel comfortable sending blood through the mail. The acceptability of various shipment methods is shown in Figure 3, with 70% respondents indicating private couriers (e.g., FedEx or UPS) to be most acceptable.

Respondents provided considerations for mailing blood samples using free text. These included need for same-day shipping, stability of biospecimens, acceptable temperature ranges, anonymous shipments with barcoding, tracking system and participant transportation. Exemplary quotes included the following:

*Excellent idea if it eliminates need to travel to clinic/laboratory draw station. Similar specimen transport has been used successfully since the 1980s and works well*.

(community member)

*[It] has recently been done in some large SARS-CoV-2 serosurveys*.

(community member)

*DNA [deoxyribonucleic acid] can be found in saliva on many envelopes that are currently used*.

(biomedical HIV cure researcher)

Perceived benefits of mailing blood samples included convenience, flexibility, not having to go to a doctor’s office and pay a co-pay, and time-savings. Perceived concerns of mailing blood samples included possible delays, damaged or lost packages, mailing errors, questionable reliability of postal system, long lines at post office and limited drop-off hours. Additional concerns were biospecimen degradation or hemolysis and biohazards and genetic materials being shipped through the mail.

### 3.5. Feedback on Communicating Viral Load Test Results

**Figure 4:**
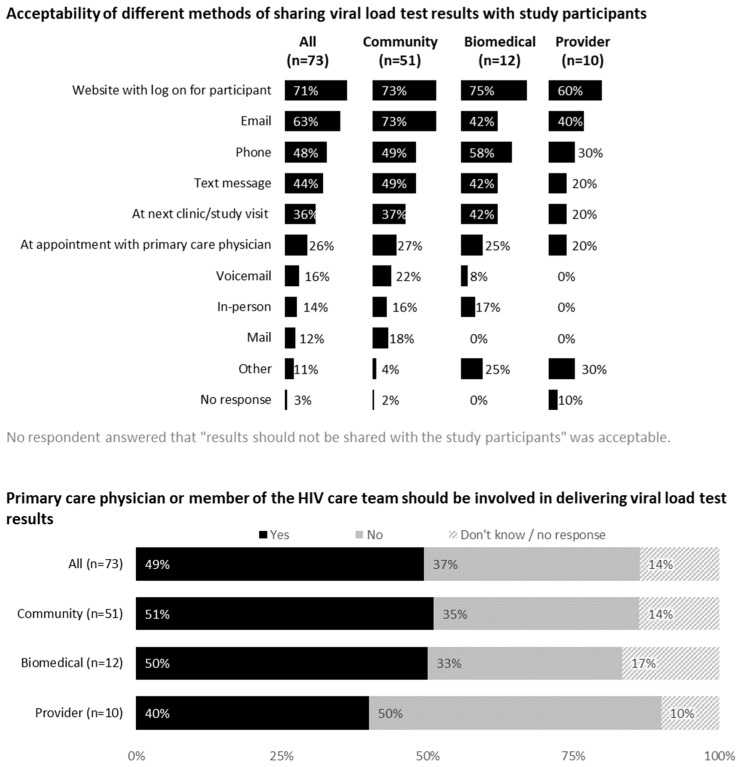
Feedback on communicating viral load test results from all participants (United States, 2021).

Acceptability of different methods for sharing viral load test results with study participants are shown in Figure 4. A confidential web portal was perceived as the most acceptable method (71% overall), followed by email (63%), phone (48%) and text messaging (44%). Respondents provided considerations for communicating viral load test results to PWH. These included text alerts informing participants when results are available online, a two-step authentication process and having participants dictate how they want results shared. Community members emphasized the need for human contact in case of questions or concerns. Free text answers included the following:

*If there is a reason for concern, test results should be shared by a real human (and a plan of action provided)*.

(community member)

*Depends on the result. If they are undetectable, can be texted or VM [voicemail]. If detectable, should be* via *phone/video chat with study team member (preferably someone with clinical knowledge) who can explain what their results mean, cause for concern or lack thereof, is participant still willing to continue their ATI,* etc. *Mail takes too long. Website may create inequity in results access. Next visit can be too long. People should have access to results in as close to real time as possible*.

(community member)

*Results should come as soon as possible since the viral load will fluctuate during an ATI*.

(biomedical HIV cure researcher)

Approximately half (49%) of the respondents indicated that primary care physicians or members of HIV care teams should be involved in communicating test results. Reasons for involving primary care teams included therapeutic relationship, monitoring and record-keeping purposes, team communication being essential during ATIs and results warranting ART restart.

*I think this would be better than just a lab tech handling it*.

(community member)

*I don’t think they need to deliver the results, but they should be notified*.

(community member)

*It’s more personal and safer than allowing patients to find out themselves electronically*.

(community member)

*PCPs [primary care providers] should always be involved if treatment is stopped for this study. The doctor may know something about the patient not known by the research members*.

(HIV care provider)

*Only if the patient becomes detectable. I feel as though emotions would need to be navigated as the patient had put their faith in the cure therapy*.

(HIV care provider)

We asked respondents to indicate any privacy or other concerns they had about the delivery of HIV viral load test results. Most respondents indicated they had no concern. Some potential concerns raised included lack of compliance with Health Insurance Portability and Accountability Act (HIPAA) requirements, difficulty with interpretation of results, participants requiring additional counseling, unauthorized access, results delivered to the wrong person and computer access or literacy issues. Exemplary quotes were as follows:

*Study Coordinator should be [the] person who calls to participants who don’t use a computer or smart phone. The rest of us should be able to login and view results. Viral rebound results should be immediately discussed with participants… Some participants would need counseling, others, like myself, would want electronic access*.

(community member)

*Difficulty with interpretation. Is a viral load of 130 [copies/mL] just a blip or does it represent the beginning of virologic failure. If testing is done frequently, this could raise a lot of questions and anxiety for patients and create additional PCP-required communication which can be challenging for providers who are already overworked and underpaid*.

(HIV care provider)

### 3.6. Feedback on Technical Support

**Figure 5:**
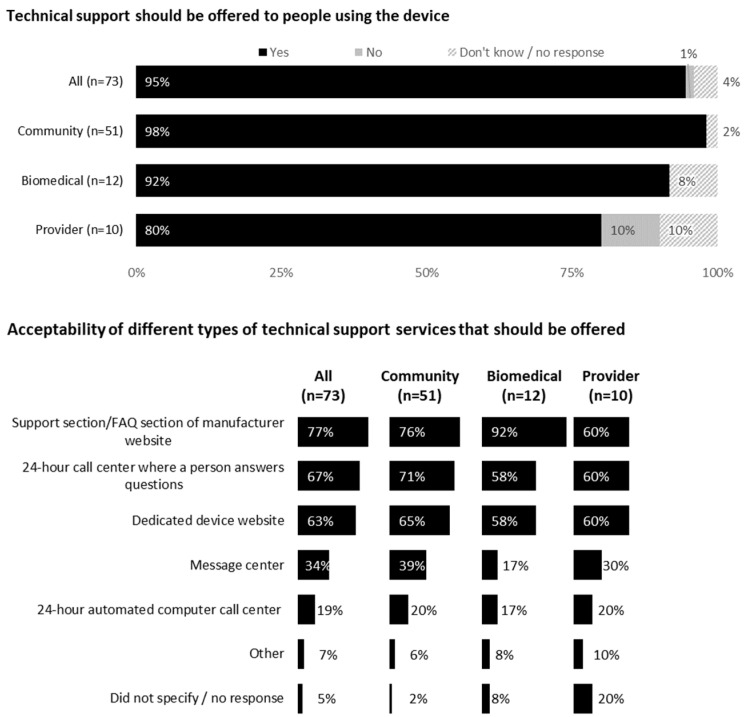
Feedback on technical support for using the device from all participants (United States, 2021).

Almost all (98% community members, 92% biomedical HIV cure researchers and 80% HIV care providers) indicated technical support should be provided with the device. Acceptability of different types of technical support services can be found in Figure 5. Most acceptable forms of technical support included support section/frequently asked questions (FAQs) on manufacturer’s website (77% overall), 24-hour call center (67%) and device website (63%). Additional forms of technical support requested by respondents included a dedicated website with integrated chat feature and lay-friendly pictorial instructions.

### 3.7. Feedback from PWH

**Figure 6:**
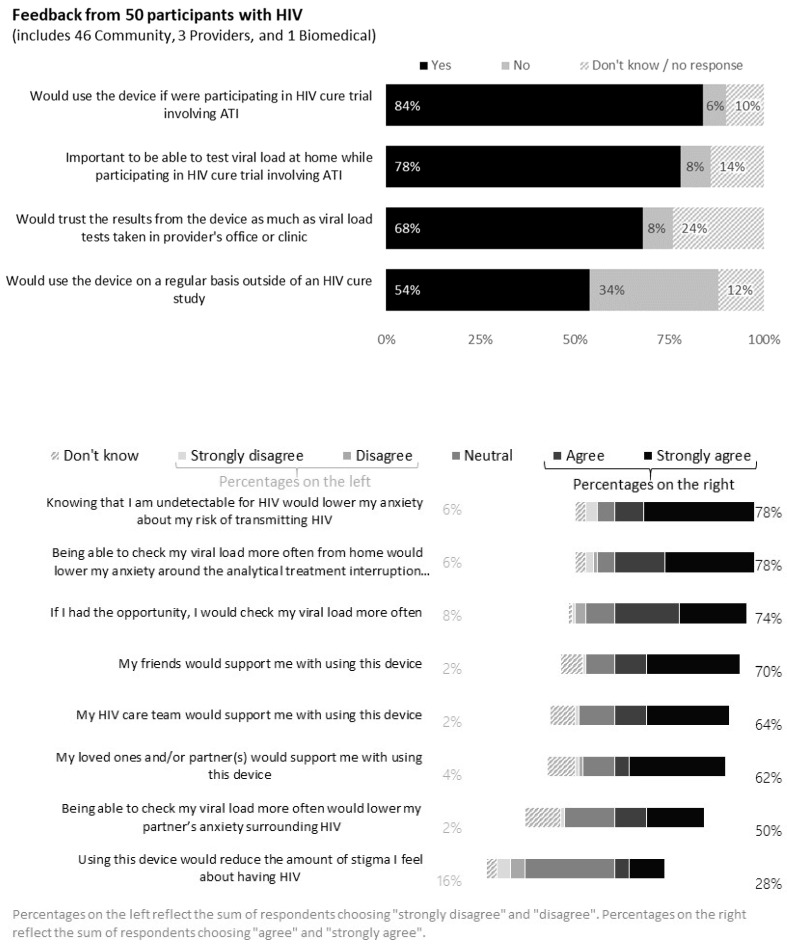
Feedback from 50 persons with HIV (United States, 2021).

Of the 50 PWH who responded to the survey, 78% indicated it would be important to be able to test viral load at home during ATI trials (Figure 6). Reasons for acknowledging the importance of home-based viral load monitoring included the need for close monitoring and timely viral load information, direct participant engagement and control, peace of mind, increased confidence in not putting oneself or partners at risk and retention of study participants during ATI trials. Free text answers included the following:

*Yes, a sharp spike in your viral load would signify the virus is multiplying and the earlier intervention, the better*.

(community member)

*I would be just as interested in the effectiveness of the study and would want immediate data in order to decide when to restart treatment if needed. Waiting for an appointment and then the results would slow information down*.

(community member)

*I suspect it will help with retention as folks are aware of what is happening with their VLs and be ready to ask more informed questions about themselves*.

(community member)

Further, 78% of PWH indicated that knowing their undetectability for HIV would lower their anxiety during the ATI. However, 8% of PWH indicated home-based blood collection for viral load testing was not important during ATI trials, because it would not obviate the need for in-clinic visits for other required tests.

Further, 84% of PWH indicated they would use the device if they were participating in an ATI trial and 68% would trust the viral load test results from blood collected from the device. Reasons for trusting the device included rigorous experimental testing, provider support and faith in the laboratory performing the testing and reporting. For example,

*The device is simply collecting the specimen, not performing the test*.

(community member)

*As long as there’s blood to test, the collection method should be irrelevant*.

(community member)

*As long as I am inform[ed] about its effectiveness and I learn it has been tested and proven to be as sensitive and effective*.

(community member)

*I’m willing to assume that the device results have been studied and compared to clinic results and have been proven to track well*.

(community member)

*I assume the samples are going to be analyzed the same way with the same quality standards as those taken in a lab*.

(community member)

Reasons for not trusting the device (8% of PWH) included concerns with accuracy and limited personalized contact and accountability.

*There is something about having personal contact at a lab that implies accountability*.

(community member)

Over half (54%) of PWH indicated they would use the device on a regular basis outside of an HIV cure study. Using the free-text option, respondents indicated they would want providers to support the use of the device. A community member cautioned that hypochondriac individuals may be challenged with the device. Sample quotes included the following:

*Until there’s a cure, it’s always good to know your VL*.

(community member)

*I think it’s awesome and it would help with stress/anxiety. We are always worried about our viral load. I’ve been undetectible [sic] for 10 years straight and I still worry about my viral load even tho I take my meds every day and know I am undetectable. It’s just scary having your livelihood rely on a med*.

(community member)

*U = U [Undetectable = Untransmittable] scares me with long times between viral load tests*.

(community member)

*In the future I could see it taking the place of lab blood draws*.

(community member)

*Definitely, please offer it to the general population outside of HIV cure studies*.

(biomedical HIV cure researcher)

However, 34% of PWH would not use the device on a regular basis outside of an ATI study. Reasons for not wanting to use the device outside of an ATI study were higher trust in clinic-based tests, comfort in current blood draw routine, need for other tests (e.g., CD4 count and chemistries) and no perceived need for testing due to sustained undetectable HIV status with current ART.

*More trust in having doc take blood*.

(community member)

*I’m comfortable with my current blood collection routine every six months*.

(community member)

*I do not feel the need to use it. I am adherent to taking my medication & my viral load is always undetectable*.

(community member)

*I have regular blood work done every 6 months and that includes tests for HIV viral load as well as several other tests. That frequency is sufficient for my HIV doctor and myself*.

(community member)

We asked respondents to provide possible additional uses of the device. These included other HIV-related measurements such as CD4 count and additional blood tests (e.g., hepatitis and sexually transmitted infections (STIs)). Respondents strongly wanted the device to become part of routine personalized clinical care and telehealth or to be used to test for viral load in case they missed an ART dose.

Respondents cited additional populations who may benefit from the device, such as individuals with difficult venous access or needle aversion, or individuals on long-acting PrEP or long-acting treatment, homeless or unstably housed individuals, or those living in rural areas. Exemplary quotes included the following:

*Conveniently verifying undetectability of viral load closer to sexual encounters & new relationships*.

(community member)

*Helpful for individuals with “tricky” veins, hardened by chemotherapy… or the ones scared of needles. It would provide anxiety relief to those who may believe that the study scheduled lab works are too few*.

(community member)

*Could be very useful I think… in addition to cure trials I wonder if it could have a role in self-testing for HIV viral load in people on long-acting PrEP, given the potential for masked, seronegative HIV infections identified in the LA [long-acting] cabotegravir trials*.

(community member)

*Diabetes, INR [International Normalized Ratio], liver/kidney function, STI testing, hormone treatment, drug levels*.

(community member)

*If the results are given to the participant in a timely manner, this would be great for HIV cure trials but also for regular monitoring. I don’t think this should be limited to HIV cure trials, please expand its use!*

(biomedical HIV cure researcher)

## 4. Discussion

Our study reveals high hypothetical preliminary acceptability of a novel home-based blood collection device for viral load testing and suggests its promise to support participation and enhance confidence in HIV cure-directed trials involving ATIs among a diverse range of stakeholders (e.g., PHW and community members, biomedical HIV cure researchers and HIV care providers). Home-based blood collection for viral load testing may present multiple advantages over clinic-based testing, such as increased sense of control, participant engagement and lowered anxiety around viral load in the body. Our survey expands the socio-behavioral scientific literature in HIV cure-directed research [6,8] by exploring how home-based blood collection for viral load testing could help reduce ATI-related barriers and burdens. Importantly, home-based blood collection for viral load testing could signify an exciting next step to enhance personalized HIV care.

Most participants expressed excitement about the device because of better control of viral load monitoring, user-friendliness and convenience. Some participants also recognized the urgency of the device in the context of ATI trials. Participants compared the device with familiar implements used to monitor other chronic diseases, such as glucometers employed in diabetes management [33]. A major perceived advantage was the possibility to escape venipunctures. However, a key notable difference is that the home-based blood collection device for viral load testing would still require sending specimens to a laboratory on the same day the sample is drawn and results would not be immediate. The device was also associated with additional possible concerns, such as user errors, lower accuracy, biohazard disposal and environmental footprint, among others. Our findings corroborate those of previous research studies on attitudes towards self-testing which showed concerns around user errors or perceived a lower accuracy of home-based tests [17,24,29,38].

An important contribution of the device noted by survey participants was the ability to detect when PWH have become viremic in the context of ATI trials. This knowledge, coupled with personalized counseling from HIV cure research teams, could help mitigate unintended transmission risks to sexual partners during ATIs [8,12,39]. Additional factors that would increase acceptability of the device included fast turnaround time for results (comparable to in-clinic viral load tests), high sensitivity of viral load test, detailed instructions for use and ability to pair the device with telehealth. In the context of regular HIV care, reducing barriers to access, such as low cost or insurance coverage, would also facilitate uptake.

Encouragingly, most participants indicated they would feel comfortable shipping blood through the mail while expressing a clear preference for private couriers. Participants provided noteworthy considerations to facilitate the mailing of samples, such as the need for same-day shipping and careful tracking of shipments. However, some concerns were noted, such as loss of anonymity/confidentiality, the possibility of mailing errors, delays and damaged or lost packages. The feasibility and advantages of sending dried blood spots by mail have been shown in previous epidemiological studies [40,41,42]. The COVID-19 pandemic has further contributed to the increased community acceptance around sending biospecimens in the form of wet blood samples through the mail [13,43].

Out of all possible options for conveying viral load test results, having a confidential web portal (protected by two factor authentication or similar security methods) was the most acceptable method. Further, easy interpretation of results, as well as availability of additional counseling if needed, were advised. The critical importance of patient–provider communication has similarly emerged as an essential consideration in the context of home-based human papillomavirus self-screening in the past [29]. Our survey findings also highlight the challenges of computer access and digital literacy for accessing the results. In a resource-limited context, a recent study highlighted the feasibility of returning viral load test results to PWH, but this required developing the necessary laboratory and data management capacity and having dedicated staff [44].

Over half of participants with HIV would use the device outside of ATIs and as part of regular HIV care. Increasing pluri-potency of the device (e.g., expanding its use to CD4 count or other regular tests) would augment acceptability, since CD4 testing is recommended once every two weeks along with weekly viral load testing during the initial weeks of ATIs [1,45]. The device was successfully utilized recently to test individuals for COVID-19 antibodies [43].

**Figure 7:**
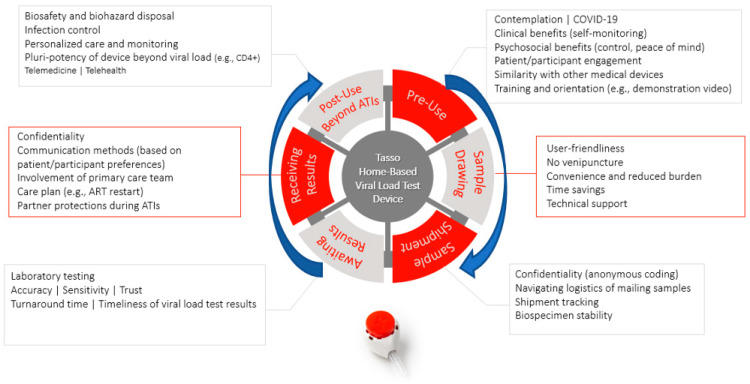
Patient/participant journey with home-based blood collection device for viral load testing.

Figure 7 summarizes the patient/participant journey with the home-based blood collection device for viral load testing based on survey findings. Supplementary Table S1 provides additional open-ended survey responses.

### Limitations

We acknowledge inherent study limitations. Our survey methodology is prone to selection and social desirability bias and was limited by its hypothetical nature with results based on perceptions rather than experiences. We have a separate (presently ongoing) study to examine actual user experiences with home-based blood collection devices for viral load testing in the context of ATIs. Additionally, due to funding and time constraints, our sample size was relatively small and skewed towards cisgender men, White/Caucasian ethnicity, older age groups and community respondents. As a result of the small and skewed sample, we were unable to conduct more complex bivariate or multivariate analyses. Our results are likely not generalizable to the broader population of stakeholders involved in HIV research and care in the United States. However, according to a recent landscape analysis of HIV cure research [46], our sample of study participants seem largely representative of community stakeholders currently involved in HIV cure research. Increased efforts would be needed to augment diversity in the field of HIV cure research [47]. We did not verify HIV status of self-reported PWH; however, we advertised the survey specifically among HIV cure research networks. We also implemented the survey with individuals directly involved in HIV cure-directed research and with robust bot control steps [35]; thus, we have assurance in the validity of the responses received, although this limited our sample size. The limited compensation may have further reduced our sample size. We were unable to recruit participants from the Midwest region of the United States (most HIV cure research is currently concentrated in the West, Northeast and South regions). Our survey was only available in English, which may have resulted in under-representation of Spanish-speaking respondents. We did not assess willingness-to-pay for the home-based blood collection device for viral load testing, as this was outside the scope of our current study. These limitations notwithstanding, our study represents, to our knowledge, the first nationwide survey to assess preliminary acceptability of a novel home-based blood collection device for viral load testing in the United States.

## 5. Conclusions

We found high acceptance of a novel home-based blood collection device for viral load testing in the context of HIV cure-directed clinical trials involving ATIs. Survey findings have implications across the HIV research and care continuum. The home-based blood collection device for viral load testing could help reduce the risk of onward HIV transmission in the context of ATI trials. For HIV treatment, the device could provide a means for better, more accessible, personalized HIV care and monitoring. For HIV cure-directed research, the device could become an important adjunct to ongoing ATI trials to alleviate participant anxiety around being off ART. The home-based blood collection device for viral load testing may represent a vital step towards patient/participant-centered ATI trials [48,49] and routine HIV clinical care. Survey findings could help inform future implementation and uptake into real-world contexts. More research will be needed to design interventions to support the successful introduction of the device in the context of both research and personalized care. Similar surveys should be implemented in resource-limited settings, where remote or home-based blood collection for viral load testing could prove even more transformative. In a global context, increasing use of home-based HIV monitoring (such as HIV antibody and viral load testing) could signify a marked impact on progress towards achieving the UNAIDS targets [50].

## Figures and Tables

**Table 1:** Demographic characteristics of survey respondents (United States, 2021).

		*n*	Full Sample %	Community %	Biomedical %	Provider %
Gender		*n = 73*		*n = 51*	*n = 12*	*n = 10*
	Cisgender man	51	70%	76%	58%	50%
	Cisgender woman	18	25%	22%	25%	40%
	Transgender man	1	1%	0%	8%	0%
	Transgender woman	1	1%	2%	0%	0%
	Gender non-binary or gender queer	1	1%	0%	0%	10%
	Gender identity not listed	1	1%	0%	8%	0%
Sex assigned at birth		*n = 73*		*n = 51*	*n = 12*	*n = 10*
	Male	53	73%	78%	58%	60%
	Female	20	27%	22%	42%	40%
Highest level of completed education		*n = 73*		*n = 51*	*n = 12*	*n = 10*
	High school diploma or G.E.D.	3	4%	4%	0%	10%
	Some college or a 2-year college degree	18	25%	35%	0%	0%
	4-year college degree	20	27%	35%	8%	10%
	Master’s degree or professional degree, or equivalent	16	22%	22%	33%	10%
	Doctorate degree or equivalent	16	22%	4%	58%	70%
Region of residency		*n = 73*		*n = 51*	*n = 12*	*n = 10*
	Northeast	22	30%	22%	67%	30%
	Midwest	0	0%	0%	0%	0%
	South	15	21%	22%	17%	20%
	West	30	41%	45%	17%	50%
	Did not specify	6	8%	12%	0%	0%
Ethnicity identity		*n = 73*		*n = 51*	*n = 12*	*n = 10*
	Hispanic or Latino/Latina/Latinx	7	10%	10%	17%	0%
	Not Hispanic or Latino/Latina/Latinx	66	90%	90%	83%	100%
Racial identity		*n = 73*		*n = 51*	*n = 12*	*n = 10*
	White or Caucasian	49	67%	69%	75%	50%
	Black or African American	13	18%	22%	0%	20%
	Asian	5	7%	0%	25%	20%
	Other	3	4%	6%	0%	0%
	More than one race	2	3%	2%	0%	10%
	Prefer not to answer	1	1%	2%	0%	0%
Age		*n = 73*		*n = 51*	*n = 12*	*n = 10*
	Mean (years)	55		60	44	42
	Median (years)	56		59	41	40
	Standard deviation (years)	13		9	15	10
	Interquartile range (years)	50–63		55–65	35–56	36–43

## Data Availability

All relevant data related to this survey are included in this manuscript.

## Supplementary Materials

The following are available online at https://www.mdpi.com/article/10.3390/jpm12020231/s1, Supplementary Table S1: Open-ended survey responses: patient/participant journey stages, key categories, summary and considerations.
