# Supplementary material for: Preliminary Acceptability of a Home-Based Peripheral Blood Collection Device for Viral Load Testing in the Context of Analytical Treatment Interruptions in HIV Cure Trials: Results from a Nationwide Survey in the United States
Source: J Pers Med. 2022 Feb 7;12(2):231. doi: 10.3390/jpm12020231 (PMC8879991; doi:10.3390/jpm12020231)
Supplement: Supplementary file 1 [file jpm-12-00231-s001.zip › jpm-1511351-supplementary.pdf]

Supplementary Table S1. Open-Ended Survey Responses: Patient/Participant Journey Stages, Key Categories, Summary and Considerations.

Journey Stage and Key Categories	Exemplary Quotes	Summary & Considerations
Pre-use: COVID-19	*This is urgently needed. Especially with COVID, frequency of in person monitoring has become a major safety issue* – Biomedical HIV Cure Researcher<br><br>*This means that participants could test from the safety of their home without having to incur the COVID risk of traveling to a research site* – Biomedical HIV Cure Researcher	Respondents identified and acknowledged the urgency of the home-based viral load test device in the context of the COVID-19 pandemic.<br><br>Respondents expressed that they would feel confident and more engaged in the research process if they would use the device.<br><br>Respondents suggested that the packaging should be neutral and should not indicate viral load testing.<br><br>Respondents also highlighted the potential need of training so that clients can use the device with ease.
Clinical benefits (self-monitoring)	*It would increase confidence that I'm not putting my health at risk.* – Community Member	
Peace of Mind	*It gives a person peace of mind and keeps them involved in the process* – Community Member	
Training and orientation	*This is not a simple process, and it would require thorough training, and may remain out of reach for some patients* – Biomedical HIV Cure Researcher	
Sample Drawing: User friendliness	*Easy to use and you do not need another medical professional to administer.* – Community Member<br>*It seem[s] easy to use and that will allow me to monitor viral load without having to visit the doctor.* – Community Member<br>*Very friendly to use.* – HIV Care Provider	Respondents noted overall that the device would be easy to use, convenient and it would save time, and in turn, improve uptake.<br><br>Respondents emphasized the need for adequate technical support.
No venipuncture	*Ease of use. At home. No venipuncture needed.* – Community Member	
Convenience and reduced burden	*Very cool and handy! Especially for people who have a hard time with transportation or have restrictions on mobility.* – Biomedical HIV Cure Researcher	
Time savings	*It's saving time and may therefore increase compliance.* – Community Member<br>*It would save time by not having to go get blood drawn.* – Community Member	
Sample Shipment: Confidentiality (anonymous coding)	*It's OK as long as it can be kept anonymous by coding the samples* – Biomedical HIV Cure Researcher<br>*To know that both the delivery and sending of the device is maintain[ed] confidential.* – Biomedical HIV Cure Researcher<br>*Loss of confidentiality, would have to not include address and would have to have study ID [identification] instead.* – HIV Care Provider	Anonymous coding is recommended to protect privacy.<br><br>Respondents expressed concerns about stability and longevity of biospecimens at various temperatures along with tracking of samples.<br><br>Respondents were comfortable mailing their samples except in settings where mailboxes or post offices would be remote or unreliable.<br><br>More information should be provided on speed of shipment to the laboratory after blood collection.<br><br>Respondents recommended a robust tracking system to track samples.
Navigating logistics of mailing samples	*Mailing the blood sample seems to be a convenient method – it could be given to mail carrier when other mail is delivered.* – Community Member	
Biospecimen stability	*How stable is the sample to changes in ambient temperature (e.g., what happens if sample freezes in winter or cooks in summer while sitting in the mailbox awaiting pickup?)* – Biomedical HIV Cure Researcher	

	Are there any temperature requirements? – HIV Care Provider<br>*No problem at all. The USPS allows blood samples to be mailed provided they are in FDA [Food and Drug Administration] approved transport packaging.* – Community Member<br>*What preserves the blood? Aren't you concerned with delivery delays or other USPS [U.S. Postal Service] processing problems (e.g., lost packages)? Can these be tracked?* – Community Member	
Shipment Tracking	*There needs to be a tracking system to know that the sample was obtained, shipped, received, resulted and reported.* – Biomedical HIV Cure Researcher	
Awaiting Results:		
Accuracy	*I would need some reliability/accuracy data first.* – Community Member<br>*Since there are other partners that are invested in the data, they would also make sure to have the device give accurate data.* – Community Member<br>*No reason not to trust the results on an approved medical device.* – Community Member<br>*If the manufacturer has validated the results alongside standard viral load tests, then I would trust this.* – Biomedical HIV Cure Researcher	We noted mixed responses on perceived trust and accuracy of the device. Most respondents would want viral load tests to be as accurate and sensitive as possible.<br><br>Resources (for example, information on prior trials providing evidence on accuracy and efficacy) should be provided to increase confidence in the accuracy of the viral load test.
Sensitivity	*As long as I am inform[ed] about its effectiveness and I learn it has been tested and proven to be as sensitive and effective.* – Community Member	
Trust	*[Would you trust this device as much as traditional viral load tests?] Yes but only if verifiable accuracy data is published?* – Community Member	
Timeliness of Viral Load Results	*If the results are given to the participant in a timely manner, this would be great for HIV cure trials but also for regular monitoring. I don't think this should be limited to HIV cure trials, please expand its use!* – Biomedical HIV Cure Researcher<br>*Want the more timely data to decide to restart treatment if needed.* – Community Member	
Receiving Results:		
Communication methods	*Depends on the result. If they are undetectable, can be texted or VM [voicemail]. If detectable, should be via phone/video chat with study team member.* – Community Member<br>*Should be participant preference and with someone available to answer questions.* – HIV Care Provider<br>*If they are elevated, a team member should speak with the patients about safety.* – Biomedical HIV Cure Researcher	Respondents noted that the method of sharing test results would depend on undetectable vs. detectable status. If the participant has an undetectable status, results could be sent via secured email or text. If detectable, there should be human involvement or contact.
Involvement of primary care team	*No need, but they [primary care providers (PCP)] should be notified if it's detectable.* – Community Member<br>*With respect to above question, PCP does not need to be involved but needs to be informed so they are aware should patient[s] have questions.* – HIV Care Provider	Some respondents noted that PCPs should be in loop while others indicated PCPs can be informed if there are concerns. Multiple options should be provided for sharing test results to meet the needs of participants and for keeping PCPs informed.
Restarting ART	*They may get faster feedback of viral resurgence, hence needing to start treatment again.* – Community Member	Respondents noted how the device could help indicate when ART restart may be needed in the context of ATI trials.

Partner protections during ATIs	*To know if I might transmit to sexual partners, and if I should restart HIV meds.* – Community Member	
Post-Use:<br>Biosafety and biohazard disposal	*It's interesting, but a lot of waste: plastic bag is huge, so is box.* – Community Member<br>*There would be blood remnants in the device. Wouldn't this be considered biohazard waste? How would we dispose of it safely?* – Community Member<br>*Proper sharps disposal, or if the device is designed so it can be disposed of in regular trash?* – HIV Care Provider	Respondents expressed concerns over biohazard waste and environmental footprint of the device.<br><br>Minimal waste, environmental-friendly packaging and clear instruction on disposal should be considered.
Infection control	*Infection from improper cleaning, bleeding in elderly.* – Biomedical HIV Cure Research	
Beyond ATIs:<br>Personalized care and monitoring	*I think it's awesome and it would help with stress/anxiety. We are always worried about our viral load.* – Community Member<br>*I still have to go in for other lab work, e.g., CD4+, so I would not use this.* – Community Member	Respondents with HIV noted they would prefer to use the device outside of ATIs to know their viral load.
Pluri-potency of the device beyond viral load	*Diabetes, INR [International Normalized Ratio], liver/kidney function, STI testing, hormone treatment, drug levels.* – Community Member<br>*Anything that can be tested by blood draws.* – Community Member	Manufacturer(s) of the device should consider increasing pluri-potency of the device (e.g., additional tests requiring blood draws) to increase its acceptability.
